# How to get a smoker addicted to quitting

**DOI:** 10.1007/s12471-016-0920-9

**Published:** 2016-11-18

**Authors:** M. E. W. Hemels

**Affiliations:** 1 Rijnstate Hospital Arnhem, Arnhem, The Netherlands; 2 Radboud University Medical Center Nijmegen, Nijmegen, The Netherlands

In the last decades, the negative effects of cigarette smoking resulted in a significant increase in chronic cardiovascular and pulmonary disease, cancer and preventable deaths. Already in the early 1960s, the ‘Dutch antismoking doctor’ Lenze Meinsma, an oncologist who performed his PhD on the health risks of smoking, warned that smoking is extremely harmful for your health [1]. However, at that time smoking was very common in the Netherlands with over 90% of men smokers. Meinsma became director of the Dutch Cancer Society (1953–1978) campaigning tirelessly against smoking, but for a long time he was ignored and seen as a ‘fanatic’.

Yet in 1971 he finally got support from four scientific associations for medical consultants: cardiologists, internists, ear, nose, and throat specialists, and lung specialists. On Christmas eve 1974 the Dutch expert centre on tobacco control STIVORO was founded, a collaboration of the Dutch Asthma Fund, Dutch Cancer Society, and the Netherlands Heart Foundation. STIVORO took the lead in launching the first government subsidised campaigns against smoking. It was not until 1982 that government health warnings on cigarette packets became obligatory and it took much longer before smoking was prohibited at work (Tobacco Act 1990) and in enclosed public spaces and buildings (Tobacco Act 2002). The proportion of male smokers started to decline from about 95% in the late 1960s to 36.7% in 1997 [2]. The proportion of female smokers initially increased from about 30% in the 1960s to beyond 40% in the 1970s and then started to decrease to its previous level, 30.3% in 1997. At that time, smoking cessation programs were still underdeveloped, and 72% of smokers motivated to quit smoking tried to stop without aid. About 79% of smokers in the group of lowest socioeconomic status (SES) appeared to be unmotivated to quit smoking compared with 51% of patients in the group of highest SES. In 2014 the prevalence of smokers in the Netherlands was still 23% (men versus women, 24% versus 22%, respectively), of whom 81% planned to quit smoking in the future [3]. Forty percent of (ex-) smokers acknowledged to have used some kind of aid, especially in the form of an electronic cigarette, and/or participated in a smoking cessation program in their most recent quit attempt.

In this issue of the Netherlands Heart Journal, Berndt et al. describe the effects of two different smoking cessation counselling interventions among 625 smokers hospitalised for coronary heart disease [4]. It was hypothesised that face-to-face counselling increases the proportion of patients who refrain from smoking among low SES patients and patients with a low motivation to quit, whereas telephone counselling would be sufficient to reduce smoking in patients with higher SES and a high motivation to quit 12 months after hospital discharge. All three studied patient groups, including the ‘usual care’ group, received nicotine patches for eight weeks. Telephone counselling was provided by counsellors from STIVORO and lasted 15 minutes per call. Face-to-face counselling lasting 30–45 minutes per session was provided by cardiac nurses who, for the purpose of the study, were qualified as smoking cessation counsellors. The primary outcome measure, continued abstinence at 12-month follow-up including biochemical validation, was reached in 26.9% of the patients in the usual care group compared with 34.7% in the telephone counselling and 33.1% in the face-to-face counselling group, respectively (χ²(2) = 3.50, *p* = 0.17). After multivariate analysis, positive differential effects on continued abstinence for both telephone and face-to-face counselling compared with usual care were found for patients with a low SES and patients with a low intention to quit, the effect being largest for face-to-face counselling (telephone counselling: OR = 3.10, 95% CI 1.32–7.31, *p* = 0.01; face-to-face counselling: OR = 5.30, 95% CI 2.13–13.17, *p* < 0.001).

In my opinion the study by Berndt et al. [4] adds important evidence for the value and optimisation of smoking cessation programs. Smoking being highly physically and mentally addictive and extremely harmful, once again highlighted the need for an intensive and combined mental and physical treatment approach in specific patient groups in o﻿r﻿d﻿e﻿r to succeed in durable smoking abstinence. One of the main lessons of this study is that intensive cessation counselling is especially worthwhile in patients with initially weak intentions to quit smoking. Although it is typically Dutch to respect the patient’s freedom of choice, the results of this study should motivate caregivers to actively discuss the importance and proven positive effects of participation in smoking cessation programs. On the other hand, a recently published study in the Netherlands Heart Journal, by Snaterse et al. [5], showed that the majority of successful quitters at 1 year stopped immediately after their acute coronary syndrome. The authors concluded that in a large group of patients who decided to quit immediately after a life-threatening event, no relapse prevention program is needed [5].

Contrary to the gradual decline in cigarette smoking, the possible consequences of the increasing use of waterpipe smoking among young people was reviewed and discussed in a previous issue of this journal [6]. Education campaigns to raise awareness and more systematic research into the possible health consequences of smoking the waterpipe were recommended, given the indication that health consequences of smoking the waterpipe are probably equally worrisome as those of cigarette smoking. In line with this, in November 2015 the Netherlands Heart Foundation, Dutch Cancer Society and the Lung Foundation Netherlands launched a new campaign especially focussed on teenagers, aimed to collectively strive for ‘a generation free of smoking’. More than ever, current and future medicine includes not only the responsibility for innovative treatments and treatment adherence, but also awareness and a major role for lifestyle modifications such as smoking and for example obesity, assigned as ‘the new smoking’ risk factor [7].
